# Determining a critical threshold for G6PD activity below which red blood cell response to oxidative stress is poor

**DOI:** 10.1186/s12936-020-03272-y

**Published:** 2020-06-17

**Authors:** Maria Swastika, Alida R. Harahap, Lydia V. Panggalo, Sri Widia A. Jusman, Ari W. Satyagraha

**Affiliations:** 1 Red Blood Cell Enzymes and Membrane Disorders Laboratory, Eijkman Institute of Molecular Biology, Jakarta, 10430 Indonesia; 2 Master Programme in Biomedical Sciences, Faculty of Medicine, Universitas Indonesia, Jakarta, 10430 Indonesia; 3 Departement of Biochemistry and Molecular Biology, Faculty of Medicine, Universitas Indonesia, Jakarta, 10430 Indonesia

**Keywords:** G6PD deficiency, Glutathione, Heterozygous women, Malondialdehyde, Oxidative stress, Diagnostic

## Abstract

**Background:**

Glucose-6-phosphate dehydrogenase (G6PD) deficiency is the most common enzyme disorder in the world. Its main function is to generate NADPH that is required for anti-oxidative pathway in the cells especially in red blood cells (RBC). G6PD deficiency is X-linked and thus subject to random X-chromosome inactivation in women giving them mosaic expression of G6PD activities in their individual cells. This phenomenon makes it difficult for diagnosis with the currently available G6PD qualitative diagnostic tests. With the rolling out of newly marketed anti-malarial drug tafenoquine, which has a long half-life, screening for G6PD deficiency becomes a necessity where those with < 70% G6PD activity cannot receive this drug. Thus, evidence for a quantitative cut-off for G6PD activity is needed to ensure safe drug administration.

**Methods:**

RBC models were developed to analyse the effect of oxidant on RBC oxidative markers namely total glutathione (GSH)and malondialdehyde (MDA). G6PD activity was measured using quantitative assay from Trinity Biotech and was correlated with cytofluorometric assay. RBC from two G6PD heterozygous women with different G6PD activities were also analysed for comparison.

**Results:**

There was a negative correlation between G6PD activity and CuCl concentration and a strong association between G6PD activities and proportion of G6PD normal RBC in CuCl-treated models and in ex vivo RBC. However, in terms of oxidative stress markers analyses, unlike the hypothesis where the lower G6PD activity, the higher MDA and the lower GSH level, the CuCl RBC model showed that in low G6PD activities (10–30%) cells, the MDA level is lower compared to the rest of the models (p < 0.05). The ex vivo models however were in line with the hypothesis, although the result was not significant (p = 0.5). There was a significant difference between RBC with < 60% and those with > 80% G6PD activities in CuCl RBC model, but not in ex vivo RBC (p = 0.5). Genotyping heterozygous subjects showed G6PDViangchan variant with 2.97 U/gHb (33% activity) and 6.58 U/gHb (74% activity).

**Conclusions:**

The GSH analysis has pointed to the 60% G6PD activity cut-off and this data is supportive of the old World Health Organization threshold for intermediate upper limit of 60% G6PD activity. However, there are significant limitations in using MDA assay with CuCl RBC model because the RBC was already stressed due to the copper treatment and thus present a different result when compared to the ex vivo model.

## Background

Glucose-6-phosphate dehydrogenase (G6PD) is a house-keeping enzyme that acts as a rate limiting enzyme in the pentose phosphate pathway. It generates NADPH which is required for downstream anti-oxidative activity in glutathione (GSH) cycle. G6PD deficiency is the most common enzymopathy in the world, affecting more than 400 million people; most are living in malaria endemic countries. G6PD deficiency is caused by a functional mutation of the enzyme, making it unstable and less able to form dimers or tetramers thus decreasing enzyme activity [1]. Clinical manifestations of G6PD deficiency vary widely from mostly asymptomatic to chronic anaemia (non-spherocytic haemolytic anaemia). The deficiency is defined according to the 1967 World Health Organization (WHO) enzyme classification. Class I as the most severe and chronically anaemia; class II as severe and class III as intermediate; and class IV as normal [2].

G6PD deficiency is an X-linked disorder and thus deficient males and homozygous deficient women will exhibit the full extent of the deficiency whereas G6PD heterozygous women will have a range of G6PD activities from deficient to very normal G6PD activity. This is due to random X-inactivation that occurs very early on in the epiblast during gastrulation in individual cells [3–6] creating genetic mosaicisms among G6PD heterozygous women with the same gene mutation [4]. These mosaicisms give rise to variable G6PD activities and are thus very difficult to diagnose with the currently available qualitative diagnostic tests. Current qualitative tests can only detect < 30% G6PD activities. Previous studies have shown that the heterozygous women with G6PD activities between 30 and 80% of normal are the most difficult to diagnose since they may give ‘normal’ results, and yet, they can still haemolyse upon exposure to oxidative agents such as drugs (anti-malarial primaquine), food (fava beans) and infections [7–9]. Single-dose tafenoquine, another 8-aminoquinoline drug, has recently been approved by FDA (United States Food and Drug Administration) as another radical cure for malaria, aside from primaquine [10]. Because of the long half-life of tafenoquine, screening for G6PD must be done prior to giving the drug to malaria patient. To avoid the potential harmful side effect of the drug to G6PD deficient individuals, those with G6PD activity ≤ 70% of normal should not receive this drug and this includes G6PD heterozygous women [11].

G6PD quantitative test is the only test capable of diagnosing G6PD heterozygous women [12]. However, the severity of haemolysis in relation to the quantitative result when exposed to oxidative stress is unknown in these women. Oxidative stress is an imbalanced condition between the level of oxidant and antioxidant. Normally, cells have the abilities to maintain the redox equilibrium of oxidant and antioxidant level [13–15]. However, the imbalance of redox equilibrium may occur in certain conditions, such as impairment in antioxidant systems leading to oxidative stress [16, 17]. Oxidative stress in red blood cells (RBC) occur when RBC are exposed to endogenous and exogenous oxidative agents in the circulation [18]. High exposure of oxidative agents to RBC will lead to the haemoglobin oxidation to methaemoglobin and thus condense into Heinz body formations. The Heinz bodies will precipitate in the RBC membrane and become attached to cytoskeletal proteins, such as spectrin, and membrane proteins, such as Band 3. This process eventually leads to RBC membrane disruption and haemolysis or removal in the spleen [19, 20].

In this study, an in vitro RBC model was employed using CuClfor G6PD deficiency as described by Baird et al. [7] that were exposed to high concentration of oxidant(300 mM H_2_O_2_) to determine the quantitative cut-off for haemolysis. The extent of oxidative stress and degree of protection from G6PD will be measured using assays such as MDA and GSH, respectively. This result was validated in ex vivo RBC from G6PD heterozygous women having the same genotype. Knowing this cut-off value likely to result in haemolysis enables the treatment of some G6PD heterozygous women who would be likely to tolerate primaquine or tafenoquine therapy for malaria who otherwise might have been excluded because of their heterozygosity.

## Methods

### Subjects and sample preparations

The RBCstudies were developed from two normal males having normal G6PD activities and normal haematological profiles and two G6PD heterozygous women having normal haematological profiles. Informed consent was obtained from all subjects prior to obtaining 8 mL of venous blood into ACD tubes from two normal males and 2 mL of venous blood into EDTA tubes from two G6PD heterozygous women. Complete blood count was performed for each subject immediately after blood collection using automated haematology analyzer Cell Dyne pocH-100i (Sysmec, USA) and the blood was aliquoted accordingly for CuCl treatment, H_2_O_2_ incubation, G6PD activity measurement, flow cytometer analyses, MDA and GSH tests and were stored at 4 °C until used. Ethical clearance for the study was approved by The Ethics Committee of The Faculty of Medicine, Universitas Indonesia (No. 640/UN2.F1/ETIK/2016).

### Red blood cells G6PD deficiencymodels using CuCl

Both Cu^+^ and Cu^2+^ can inhibit G6PD activity in RBC [21, 22]. Slight modification to the method previously described was made [7]. Procedures were done as soon as blood was collected where 400 µL of whole blood in ACD was treated with 10 mM CuCl in water (Fluka, Germany) in various final concentrations of 0 mM, 0.2 mM, 0.4 mM, 0.6 mM, 0.8 mM, 1.0 mM, 1.5 mM and 2.0 mM CuCl to mimic 10%, 20%, 30%, 40%, 50%, 60%, 70%, 80% and 100% G6PD activities in heterozygous women, respectively. All samples were incubated in a 37 °C water bath for 24 h without shaking.

### G6PD quantitative test

G6PD activity was measured for each sample after treatment with CuCl using quantitative test from Trinity Biotech (Trinity Biotech, Ireland) to determine the enzyme activity. The assay measured the rate of reduction of NADP^+^ to NADPH spectrophotometrically at 340 nm using ultraviolet spectrophotometer (Shimadzu UV–VIS 800, Japan). The rate of NADPH formation is proportional to G6PD activity. Haemoglobin was measured using HemoCue (Hemocue^®^ Hb 301 System, Sweden). G6PD activity is calculated in relation to haemoglobin level in U/g Hb according to the manufacturer’s manual.

### H_2_O_2_ oxidative stress treatment

Prior to incubation with H_2_O_2_, the RBC previously treated with CuCl, as well as the RBC from heterozygous G6PDsubjects and normal controls, were washed twice with phosphate buffer pH 8.0. Plasma and buffy coat were discarded during the washing steps. After the first wash, complete blood count was performed for all samples to calculate the amount of RBC in each sample. Final volume of 200 µL of RBC suspension from each sample (each sample contained 10^8^ RBC/200 µL suspension) was incubated with H_2_O_2_(30%, Merck, Germany) to final concentration of 300 mM at 37 °C for 2 h, to induce oxidative stress in these cells. These would then be measured for glutathione and TBARS assays. Meanwhile, H_2_O_2_-untreated RBC from G6PD heterozygous subjects and G6PD normal controls were also incubated at 37 °C for 2 h alongside treated RBC samples.

### Malondialdehyde measurement assay

Lipid peroxidation is an indicator of cellular damage caused by oxidative stress and MDA is a good marker of such lipid peroxidation. To measure MDA, OxiSelect TBARS Assay (Cell Biolabs, USA, Cat.# STA-330) was used where thiobarbituric acid reactive substances (TBARS) is a rapid and direct quantitative measurement of MDA in biological samples. MDA forms a 1:2 adduct with TBA and can be measured colorimetrically. The procedure followed the manufacturer’s protocol where an additional step was added to get rid of the interfering haemoglobin and its derivatives by adding *n*-Butanol. All samples and MDA standards were transferred to cuvettes including a blank control and read at 532 nm absorbance. All samples and standards were done in duplicates. All MDA standards were plotted into a standard curve and MDA concentration for every sample was measured in pmol/µg total protein where the protein was measured according to Lowry protein quantitation method [23].

### Glutathione assay

Reduced GSH, as the major anti-oxidant in cells, especially in RBC, is normally present around 90–95% of total glutathione in the cells. Intracellular level of GSH is used as an indicator of the overall redox state of the cell. In normal cells, increased level of GSH indicates that there is an oxidative pressure within the cell. The Glutathione Assay kit (Sigma-Aldrich, Germany, Cat.# CS0260) provides the means to measure level of total glutathione (GSSG and GSH) in a biological sample. The kit uses a kinetic assay where the catalytic amounts of GSH cause a continuous reduction of 5,5′-dithiobis(2-nitrobenzoic acid) (DTNB) to TNB and the generating GSSG is recycled by glutathione reductase and NADPH. The rate of reaction is proportional to the concentration of GSH up to 2 µM where the yellow product, TNB is measured spectrophotometrically at 405 nm and level of GSH is measured in nmol/mg of protein in the sample as stated in the manufacturer’s protocol with slight modification, wherein a 405 nm wavelength was used instead of 412 nm.

### G6PD cytofluorometric assay

Prior to treatment with H_2_O_2_, percentage of G6PD deficient cells were analysed cytochemically to see whether they coincided with G6PD activities. The assay involved nitrite which oxidized all oxyhaemoglobin to methaemoglobin. The methaemoglobin was reduced back to oxyhaemoglobin by glucose as substrate and Nile blue as redox catalyst. This reaction depended on NADPH and G6PD normal RBC would have oxyhaemoglobin converted faster than G6PD deficient RBC. The addition of cyanide reacted with methaemoglobin and produced cyan-methaemoglobin, while oxyhaemoglobin remained inactive. Afterward, H_2_O_2_ introduction generated fluorescence only in oxyhaemoglobin thus distinguishing G6PD normal from G6PD deficient RBC. Fifty µL of packed RBC was mixed with 90 µL of phosphate buffer pH 8.0 for model cells and only 10 µL of packed RBC and 90 µL of phosphate buffer pH 8.0 for G6PD heterozygous subjects prior to applying the protocol from Shah et al. where the assay assessed G6PD activity at the level of individual RBC [24]. The samples were analysed in BD Acuri C6 + cytometer (BD Biosciences, USA), using setting system 10,000 total events with FSC-H > 30,000 collected. Samples were excited using an Arion laser (488 nm) and fluorescence emission was measured in the FL1 channel (533 ± 30 nm).

### DNA extraction and genotyping

DNA of G6PD heterozygous subjects were extracted using Wizard Genomic Purification DNA Kit (Promega, USA, Cat.# A1120) with a small modification, whereas DNA isolated from 300 µL of whole blood was diluted in 50 µL of DNA Rehydration Solution. DNA was PCR amplified and cut at various restriction enzyme sites to detect for various known G6PD variants that are common in Indonesia [25, 26]. The fragments of PCR/RFLP product were then analysed using agarose gel electrophoresis.

### Statistical analysis

To measure the correlation between CuCl concentration and G6PD activities, we used Spearman test. Linear regression test was used to analyse the association between G6PD activities with proportion of normal RBC in RBC models. Wilcoxon test was used to test the significance between RBC models in MDA and G6PD activities, as well as MDA and GSH levels in heterozygous G6PD subjects. Meanwhile, Kruskal–Wallis test was performed to analyse the differences between GSH and G6PD activity in RBC models. The data analysis was completed using RStudio version R i386 3.3.1. (http://www.rstudio.com).

## Results

### DNA genotyping of G6PD heterozygous samples

Various G6PD variants that are common in Indonesia were screened in two female subjects in this study. PCR/RFLP results from the 2 subjects having deficient and normal G6PD activities (33% and 74%, respectively) showed that they were both heterozygous for Viangchan variant (871G > A) which fall into Class II according to WHO enzyme classification. G6PD Viangchan is a severely deficient variant [26].

### G6PD activities in CuCl RBC G6PD deficiency models and G6PD heterozygous subjects

**Fig. 1:**
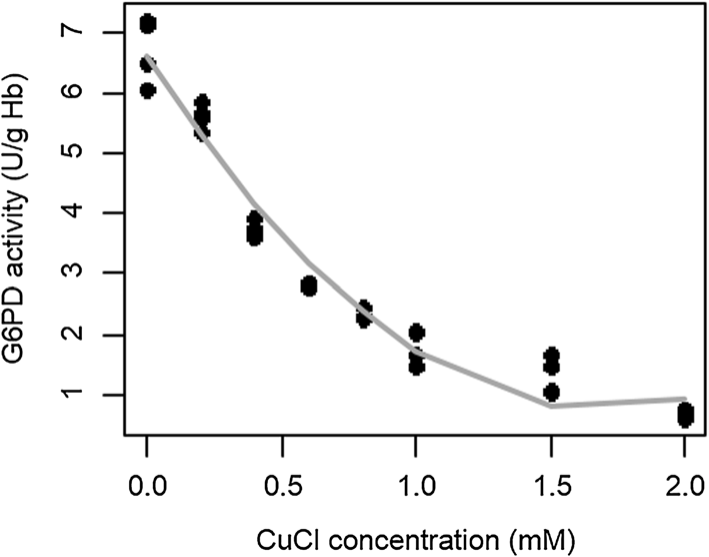
Correlation between CuCl concentration and G6PD activity in RBC models (p < 0.001; Spearman test)

G6PD activities from samples treated with different CuCl concentrations were measured quantitatively using Trinity Biotech G6PD quantitative test as seen in Table 1. The value of G6PD activity at 0 mM CuCl was set to 100% and the rest of the samples followed from 80 to 10% G6PD activities from 0.2 mM to 2 mM CuCl, respectively. The mean value for 100% and 10% G6PD activity in CuCl RBC models were 6.72 ± 0.48 U/g Hb and 0.68 ± 0.06 U/g Hb, respectively. The higher the Cu^+^, the lower the G6PD activity, as seen in Fig. 1, with *p* value less than 0.001 where the association is negatively correlated. G6PD activities in the 2 G6PD heterozygous subjects were 2.97 U/g Hb (33% activity) and 6.58 U/g Hb (74% activity). One was considered a G6PD deficient whereas the other was considered normal according to the G6PD gold standard quantitative test even though both had the same genetic polymorphism. Both subjects had normal haemoglobin level (12.6 g/dL and 13.1 g/dL) and normal complete blood count.

**Table 1:** G6PD enzyme activity and proportion of normal RBC in CuCl-treated RBC model

CuCl concentration (mM)	G6PD activity^a^ (U/g Hb)	G6PD activity (%)	Normal RBC (%)
0	6.72 ± 0.48	100	100
0.2	5.62 ± 0.18	80	89
0.4	3.77 ± 0.11	60	43
0.6	2.81 ± 0.02	50	34
0.8	2.34 ± 0.07	40	26
1	1.81 ± 0.25	30	25
1.5	1.30 ± 0.27	20	11
2	0.68 ± 0.06	10	12

^a^Data was presented in mean  ± SD

### Proportion of normal RBC in RBC models and G6PD heterozygous subjects

**Fig. 2:**
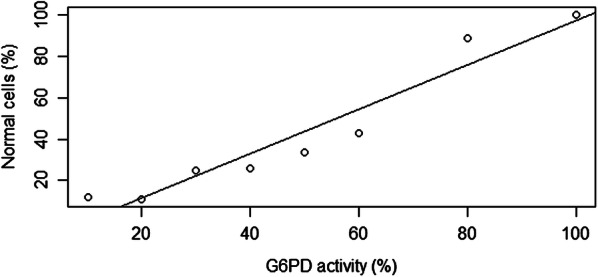
Association between G6PD activities with proportion of normal RBC in CuCl RBC model detected by cytofluorometric assay (r^2^ = 0.92, p < 0.001, linear regression test)

**Fig. 3:**
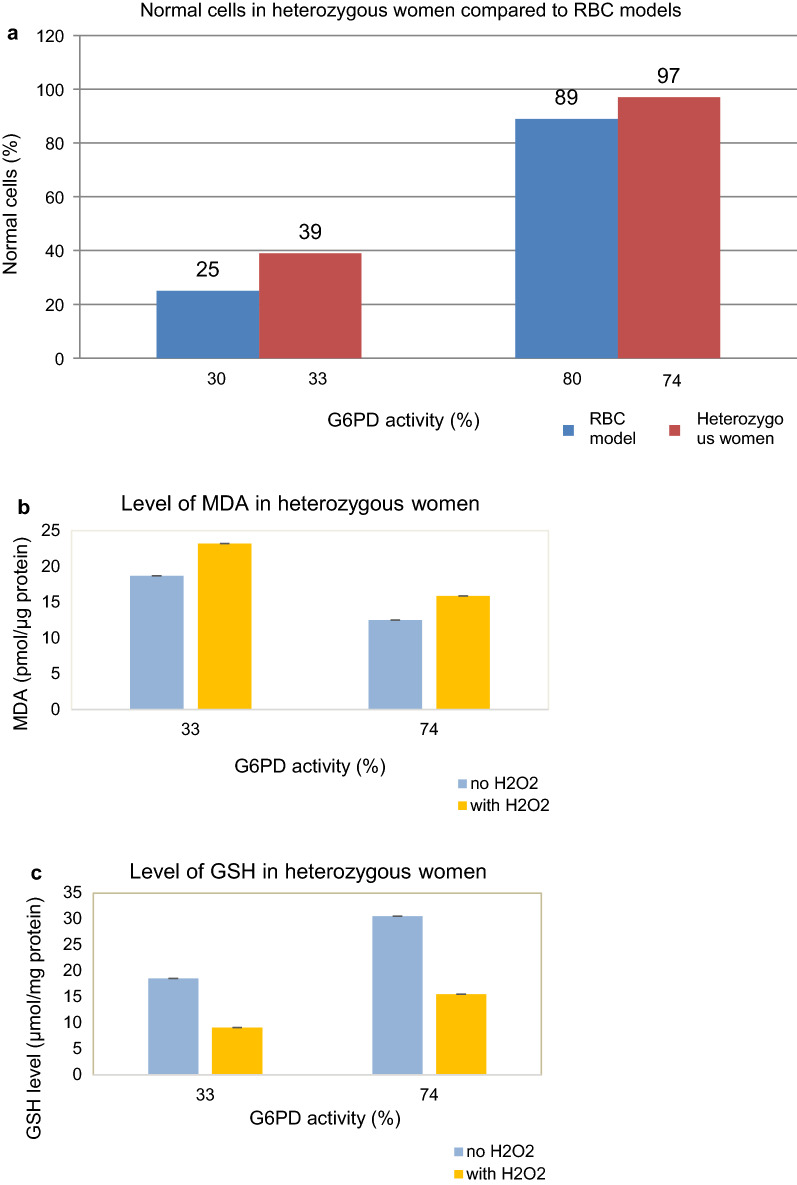
Data analysis in G6PD heterozygous subjects. **a** Comparison between proportion of normal RBC in two different G6PD activities in heterozygous women without H_2_O_2_ and RBC models with closest G6PD activities. **b** Level of MDA in two different G6PD activities in heterozygous women with and without H_2_O_2_ (p = 0.5, Wilcoxon test). **c** Level of GSH in two different G6PD activities in heterozygous women with and without H_2_O_2_ (p = 0.5, Wilcoxon test). Subject 1 has 33% G6PD activity and Subject 2 has 74% G6PD activity

The proportion of normal RBC (cells with normal activity of G6PD) in models and in G6PD heterozygous subjects were analysed cytochemically by flow cytometer. In RBC models from lowest to highest CuCl concentrations (0–2 mM), the proportion of normal RBC were 100, 89, 43, 34, 26, 25, 11 and 12% consecutively (Table 1). The results showed that the proportion of normal cells were decreased continuously with increasing CuCl concentrations, except for CuCl-treated RBC model for G6PD with 10% activity, which has a slightly increased proportion of normal RBC compared to model with 20% activity. There was a strong association between G6PD activities and proportion of normal RBC inCuCl-treated RBC model analysed using linear regression test (r^2^ = 0.92; p < 0.001) (Fig. 2). Those that were below 30% G6PD activity had only 25% normal cells compared to those with 80% G6PD activity with 89% normal cells. Interestingly, those with 60% G6PD activity had only 43% normal cells. The proportions of normal RBC were 39%in subject with 33% G6PD activity, and 97% in subject with 74% G6PD activity (Fig. 3a). There was no significant difference (p = 0.5) when the normal cells in heterozygous subjects were compared to the RBC models with the closest G6PD activities (Fig. 3a).

### Oxidative stress markers analyses

**Fig. 4:**
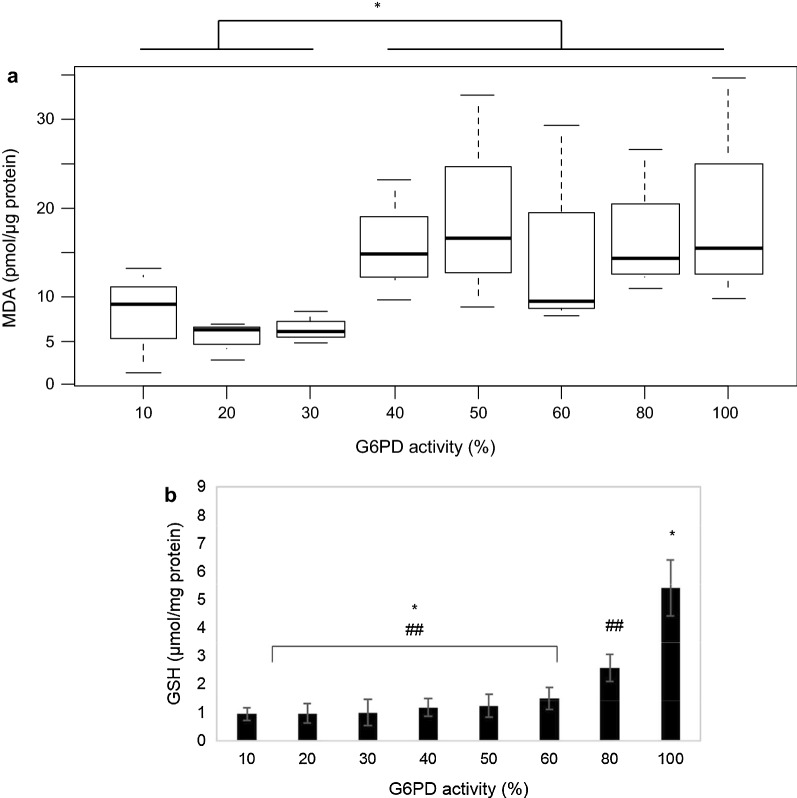
Responses of H_2_O_2_ in RBC Models. **a** Level of MDA in varying G6PD activity in RBC model. Significant difference was observed between model ≤ 30% G6PD activities with model > 30% G6PD activities (p < 0.05, Wilcoxon test). **b** GSH level in RBC models. Significant differences were observed between models ≤ 60% G6PD activities with models 80% and 100% G6PD activities (p < 0.05, Kruskal–Wallis test). * and ## denote significant groups

To analyse the extent of membrane damage in RBC, MDA was used to measure lipid peroxidation in RBC exposed to H_2_O_2_. The level of GSH was also measured to determine the level of oxidative stress in the RBC. Table 2 showed the result of MDA and GSH levels in CuCl-treated RBC G6PD models, in G6PD heterozygous women and in the G6PD normal controls. Theoretically, the lower the G6PD activity, the higher the MDA and the lower the GSH level because G6PD deficient RBC could not protect itself against oxidative damage. However, as shown in Fig. 4a, in CuCl-treated RBC G6PD models, this was not the case. It showed that low G6PD activities (10–30%) had comparatively lower MDA level compared to the rest of the models (p < 0.05).

**Table 2:** Level of MDA and GSH in CuCl-treated RBC model and in G6PD heterozygous women

G6PD activity (%)	MDA (pmol/µg protein)	GSH (µmol/mg protein)
RBC model^a,b^		
10	9.05 (1.31–13.23)	0.95 ± 0.22
20	6.27 (2.87–6.80)	0.97 ± 0.35
30	6.12 (4.84–8.35)	1 ± 0.47
40	14.78 (9.56–23.20)	1.18 ± 0.30
50	16.63 (8.88–32.72)	1.24 ± 0.31
60	9.53 (7.88–29.41)	1.49 ± 0.40
80	14.24 (10.88–26.64)	2.59 ± 0.48
100	15.41 (9.71–34.60)	5.42 ± 0.99
Controls^c^		
Normal-no H_2_O_2_	7.52 ± 2.87	13.76 ± 1.26
Normal-H_2_O_2_	15.18 ± 0.32	11.05 ± 0.17
Subjects^c^		
Subject 1-no H_2_O_2_	18.71 ± 3.92	18.6 ± 0.38
Subject 1-H_2_O_2_	23.21 ± 0.56	9.11 ± 0.01
Subject 2-no H_2_O_2_	12.52 ± 2.72	30.54
Subject 2-H_2_O_2_	15.89 ± 0.68	15.51 ± 0.24

^a^Data of MDA was presented in median (minimum–maximum value)

^b^Data of GSH was presented in mean ± SD

^c^Data of MDA and GSH were presented in mean ± SD

In the ex vivo result of the G6PD heterozygous RBC showed the expected result where MDA level is higher in G6PD deficient RBC compared to normal G6PD activity in H_2_O_2_ treated RBC (Fig. 3b), although the result was not significantly different in both subjects (p = 0.5). Comparing the ex vivo RBC between H_2_O_2_ treated and not treated cells, and deficient versus normal G6PD activity, the pattern was similar in that untreated cells have slightly less MDA compared to treated in both groups. However, the G6PD deficient RBC showed higher MDA compared to normal RBC in both treated and untreated RBC cells.

Figure 4b showed a progressively increased GSH level from very deficient G6PD RBC to very normal G6PD RBC in CuCl RBC models. There was a significant difference between RBC with < 60% G6PD activity and those with 80% and 100% (p < 0.05). Similar result was found in heterozygous women in which subject with 33% G6PD activity has lower GSH level than subject with 74% G6PD activity (Fig. 3c). Looking at the total GSH level from both groups, G6PD deficient and G6PD normal RBC, the increase of GSH level is almost twice in normal compared to deficient RBC. However, there was no significant difference of GSH level between G6PD deficient and G6PD normal RBC (p = 0.5) in ex vivo RBC. The same was observed in H_2_O_2_ untreated samples.

## Discussion

Currently, the readily available qualitative G6PD tests can only detect < 30% G6PD activity, whereas those with activities between 30 and 80% can only be determined using quantitative G6PD test. This covers women who are G6PD heterozygous. However, the need to define a cut-off value within this 30–80% G6PD activity is needed to differentiate between severe from non-severe haemolysis when exposed to oxidant.

The CuCl RBC model was developed to study the effect of oxidative stress on RBC membrane by subjecting the cells to 300 mM of H_2_O_2_ and then tried to validate the results with RBC taken from G6PD heterozygous women with the same mutation (G6PD Viangchan) but different G6PD activities where one was deficient and the other was normal according to G6PD quantitative test. Women with 74% G6PD activity would definitely be considered as normal if tested with the readily available G6PD qualitative tests. The cytochemical results positively correlated with G6PD activitiesin the ex vivo model, and thus this model can be used for oxidative stress analyses. The ex vivo RBC showed promising result that may have reflected the expected result when exposed to high concentration of oxidant unlike RBC model that were treated with CuCl prior to exposing them to H_2_O_2_.

In the RBC model treated with varying concentrations of CuCl to achieve different percentages of G6PD activities, the higher the enzyme activity, the higher the percentage of normal red cells (Fig. 2) as predicted [7]. However, there was a significant drop as seen in Table 1, from 80% G6PD activity with 89% normal cells to 60% G6PD activity with 43% normal cells (p > 0.05, was considered insignificant). It had been known that intracellular free copper was probably associated with macromolecule structures, such as DNA, enzymes and protein [27]. The over dosage of copper is toxic to cells because it interferes with glycolytic enzymes, such as hexokinase and phosphofructokinase 1, as well as other enzymes, such as phosphoglyceric kinase, 6-phosphogluconate dehydrogenase, catalase and glutathione peroxidase [21, 22, 28].

Excess copper also induces peroxidative damage in cell membranes leading to lipid bilayer destruction [29], which might explain the discrepancy of normal cells between the 80% and 60% G6PD activity. Therefore, between 60 and 80% of G6PD enzyme activity, the drop in normal RBC is significant and thus may represent the targeted cut-off. The result of the cytochemical analysis of CuCl RBC model above was validated in cytochemical analysis of *ex vivo*RBC. The ex vivo RBC showed subject with 33% G6PD activity had only 39% normal cells compared to subject with 74% G6PD activity with 97% normal cells (Fig. 3a) which might indicate that those below 60% may have already lower number of normal cells compared to those > 70% as shown in RBC model (Table 1).

MDA represents the level of RBC membrane damage caused by oxidative stress, therefore, the lower the G6PD activity, one would expect the higher the damage and thus higher MDA level. While ex vivo RBC showed higher MDA level in G6PD deficient cells compared to normal (p = 0.5, was considered insignificant), the CuCl RBC model showed the opposite, i.e. lower G6PD activity and lower MDA level [22, 30]. This could be explained as before, that CuCl already caused oxidative stress prior to H_2_O_2_ treatment of the RBC. During the CuCl procedure, the membrane had gone through peroxidative damage already, leaving behind less membrane lipid bilayer prior to H_2_O_2_ treatment. Thus, fewer MDA level was detected in the CuCl-treated RBC model. This was skipped in ex vivo RBC because there was not CuCl treatment prior to H_2_O_2_ exposure.

Glutathione cycle depended on NADPH which was generated by G6PD in RBC [31, 32]. In G6PD deficient RBC where NADPH was reduced, the total GSH was also reduced compared to normal RBC. The total GSH would be very low so that it would not overcome oxidative stress either from CuCl or H_2_O_2_. On the contrary, in normal G6PD RBC, in the presence of oxidative stress, total GSH would be induced because there was enough NADPH produced by G6PD. These results also indicated an inversely proportional correlation between CuCl and GSH level in CuCl RBC model. Rafter showed that leukocytes incubated with high concentration of Cu^2+^ decreased the reduced GSH level [33]. Previous reports by Smith et al. and Kachur et al. showed that the thiol group in GSH can be oxidized directly by copper (Cu^2+^) which led to the decrease of GSH level intracellularly and thus interfere with glutathione peroxidase function in neutralizing H_2_O_2_ to water [34, 35].

Total GSH measurements from both CuCl RBC model and ex vivo RBC showed that G6PD normal samples were higher than G6PD deficient samples (Figs. 3c and 4b). Figure 4b showed that in RBC with 80 and 100% G6PD activity there was a significant increase of total GSH to almost double and 4 times, respectively, compared toRBC with less than 60% G6PD activity. This suggested that cells with less than 60% G6PD activity did not have enough NADPH to significantly increase total GSH level. Therefore, this might suggest a cut-off (at 60% G6PD activity) in which one can use to differentiate one that is dangerously haemolytic from one that is safe when exposed to oxidant. In ex vivo RBC, there was a 30% difference of total GSH level between subject with 33% G6PD activity and subject with 74% G6PD activity, and the same for both H_2_O_2_ treated and untreated samples which suggested that the extra 30% GSH level was protective toward oxidative damage compared to those at lower G6PD activity as shown in MDA result. Figure 3b showed that the lower G6PD activity the higher MDA level as a marker for membrane damage. These findings were supported by other studies which explained that GSH level in normal RBC was higher than G6PD deficient-RBC [36–38].

## Conclusions

The GSH studies have pointed to the 60% G6PD cut-off for severe haemolysis. However, there were limitations to this study in that more G6PD heterozygous women samples, preferably with more range of G6PD activities, were needed to be analysed to validate the G6PD deficient RBC model results. The authors admit that measuring total GSH might not be as accurate as measuring reduced GSH to determine whether oxidative stress did reduce the number dramatically in G6PD deficient cells. Haemolysis assay should be done for both in vitro and ex vivo models. From the results shown so far, it is safe to conclude that CuCl does have toxic effect on RBC on top of the overdose of H_2_O_2_ in CuCl RBC model and ex vivo RBC and even more so in G6PD deficient RBC and thus a major limitation in this study.

## Data Availability

All data generated or analysed during this study are included in this published article.

## Abbreviations

CuCl: Copper chloride
G6PD: Glucose-6-phosphate dehydrogenase
GSH: Total glutathione
Hb: Haemoglobin
MDA: Malondialdehyde
NADPH: Nicotinamide adenine dinucleotide phosphate
RBC: Red blood cells
